# GTDB: an ongoing census of bacterial and archaeal diversity through a phylogenetically consistent, rank normalized and complete genome-based taxonomy

**DOI:** 10.1093/nar/gkab776

**Published:** 2021-09-14

**Authors:** Donovan H Parks, Maria Chuvochina, Christian Rinke, Aaron J Mussig, Pierre-Alain Chaumeil, Philip Hugenholtz

**Affiliations:** The University of Queensland, School of Chemistry and Molecular Biosciences, Australian Centre for Ecogenomics, QLD 4072, Australia; The University of Queensland, School of Chemistry and Molecular Biosciences, Australian Centre for Ecogenomics, QLD 4072, Australia; The University of Queensland, School of Chemistry and Molecular Biosciences, Australian Centre for Ecogenomics, QLD 4072, Australia; The University of Queensland, School of Chemistry and Molecular Biosciences, Australian Centre for Ecogenomics, QLD 4072, Australia; The University of Queensland, School of Chemistry and Molecular Biosciences, Australian Centre for Ecogenomics, QLD 4072, Australia; The University of Queensland, School of Chemistry and Molecular Biosciences, Australian Centre for Ecogenomics, QLD 4072, Australia

## Abstract

The Genome Taxonomy Database (GTDB; https://gtdb.ecogenomic.org) provides a phylogenetically consistent and rank normalized genome-based taxonomy for prokaryotic genomes sourced from the NCBI Assembly database. GTDB R06-RS202 spans 254 090 bacterial and 4316 archaeal genomes, a 270% increase since the introduction of the GTDB in November, 2017. These genomes are organized into 45 555 bacterial and 2339 archaeal species clusters which is a 200% increase since the integration of species clusters into the GTDB in June, 2019. Here, we explore prokaryotic diversity from the perspective of the GTDB and highlight the importance of metagenome-assembled genomes in expanding available genomic representation. We also discuss improvements to the GTDB website which allow tracking of taxonomic changes, easy assessment of genome assembly quality, and identification of genomes assembled from type material or used as species representatives. Methodological updates and policy changes made since the inception of the GTDB are then described along with the procedure used to update species clusters in the GTDB. We conclude with a discussion on the use of average nucleotide identities as a pragmatic approach for delineating prokaryotic species.

## INTRODUCTION

The Genome Taxonomy Database (GTDB) was developed to provide a phylogenetically consistent bacterial and archaeal taxonomy which can accommodate isolate genomes and the tens of thousands of metagenome-assembled genomes (MAGs) now being obtained from environmental and clinical samples (1–3). In order to taxonomically organise this large and growing genome dataset, the GTDB uses relative evolutionary divergence (RED) to delineate higher-rank taxa and average nucleotide identity (ANI) to delineate species clusters (4,5). Use of these quantitative criteria for circumscribing taxa allows for automated classification of new genomes (6), ensures all genomes are classified from species to domain, and normalizes the definition of taxonomic ranks across the bacterial and archaeal domains.

GTDB builds upon a number of existing public resources in order to provide a taxonomic resource that reflects recently proposed taxa, changes in taxonomic opinion, and the wealth of publicly available genomes. The NCBI Taxonomy database (7) aids in the discovery of newly proposed taxa, provides initial species assignments and specifies co-identical strain identifiers for genomes in the NCBI Assembly database (8). We currently use the NCBI Assembly database as the sole genome repository for the GTDB as it is a member of the International Nucleotide Sequence Database Collaboration (INSDC; 9) which ensures it also contains genomes submitted to DDBJ (10) and EMBL-EBI (11). The LPSN (List of Prokaryotic names with Standing in Nomenclature) database (12) is used to establish co-identical strain identifiers for the type strains of species and subspecies, the types of higher-rank taxa, and the nomenclatural status of newly proposed or reclassified taxa. The Living Tree Project (13) is used to classify 16S rRNA sequences and help resolve ambiguity in regard to the correct classification of genomes.

The latest version of the GTDB is R06-RS202 which was released in April, 2021. This is the sixth release (i.e. R06) of the GTDB since its inception in November 2017 and comprises genomes in the NCBI Assembly database as of September 2020, i.e. the release date of RefSeq 202 (14).

## RESOURCE CONTENT

### Growth of GTDB

**Figure 1.**
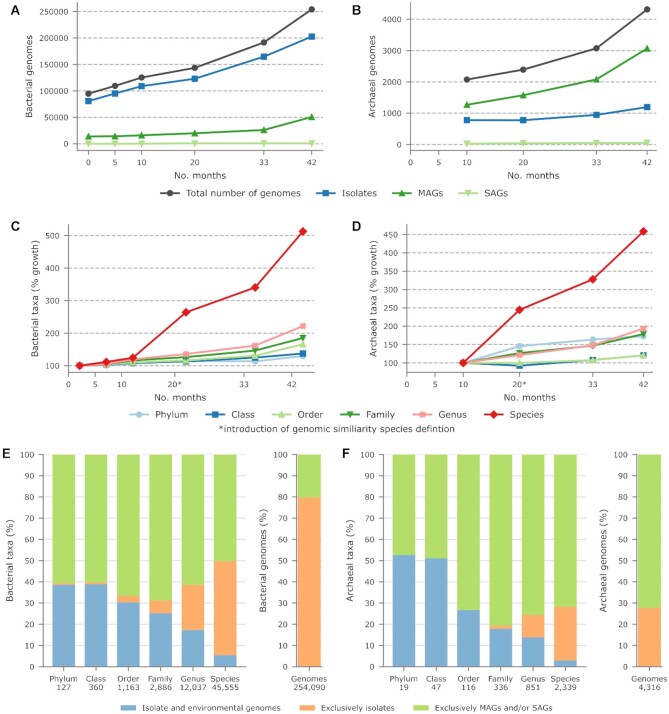
Growth of the GTDB since its inception in November 2017. (**A**, **B**) Number of bacterial and archaeal isolates, MAGs, and SAGs in the GTDB along with the total number of genomes. Archaea were introduced into the GTDB starting with R03-RS86 in August, 2018. (**C**, **D**) Percent growth in the number of bacterial and archaeal taxa in the GTDB. (**E**, **F**) Proportion of bacterial and archaeal taxa at each taxonomic rank in GTDB R06-RS202 comprised exclusively of environmental genomes (MAGs and/or SAGs), exclusively of isolates, or both isolate and environmental genomes. For comparison, the proportion of isolate and environmental genomes is shown in the right bar plot.

The number of genomes in the GTDB has grown by over 270% since its inception in November 2017 (Figure 1A and B; Supplementary Table S1). Perhaps surprisingly, bacterial isolates account for the majority of this growth rather than MAGs (or single amplified genomes; SAGs) despite recent metagenomic studies recovering tens of thousands of MAGs (2,3,15). This is a result of both the large numbers of genomes from human pathogens deposited in INSDC repositories (Supplementary Table S2) and many metagenomic studies providing only a representative MAG per operational species cluster or no MAGs to an INSDC repository. Unfortunately, this means INSDC repositories are missing much of the strain diversity which has recently been discovered. Interestingly, this pattern is not observed for archaea where MAGs are responsible for the majority of additional genomes (Figure 1B), perhaps reflecting both challenges in cultivating archaeal strains and the lack of archaeal human pathogens.

Growth in the number of genomes has coincided with a large increase in the number of bacterial (513%) and archaeal (459%) species in GTDB (Figure 1C and D; Supplementary Table S1). Importantly, we implemented an ANI-based method for delineating species starting with R04-RS89 (June 2019) which allows all genomes to be classified at the rank of species and provides a uniform estimate of species-level diversity within the GTDB (5). As expected, growth in the number of higher-rank taxa has been more modest (Figure 1C and D) though still appreciable, with the number of bacterial and archaeal genera increasing by 222% and 193%, respectively.

Unlike the number of bacterial genomes, MAGs account for the majority of taxonomic diversity covered by GTDB R06-RS202 (Figure 1E and F; Supplementary Table S1). Over 50% of bacterial taxa, regardless of rank, consist exclusively of MAGs and/or SAGs despite MAGs (50 669 genomes) and SAGs (745 genomes) representing only 20.2% of the 254 090 bacterial genomes. Similarly, nearly 50% of all archaeal taxa, regardless of rank, consist exclusively of MAGs and/or SAGs with over 70% of archaeal species, genera, families, and orders lacking an available cultured representative (Figure 1F).

### Changes to the GTDB Website

**Figure 2.**
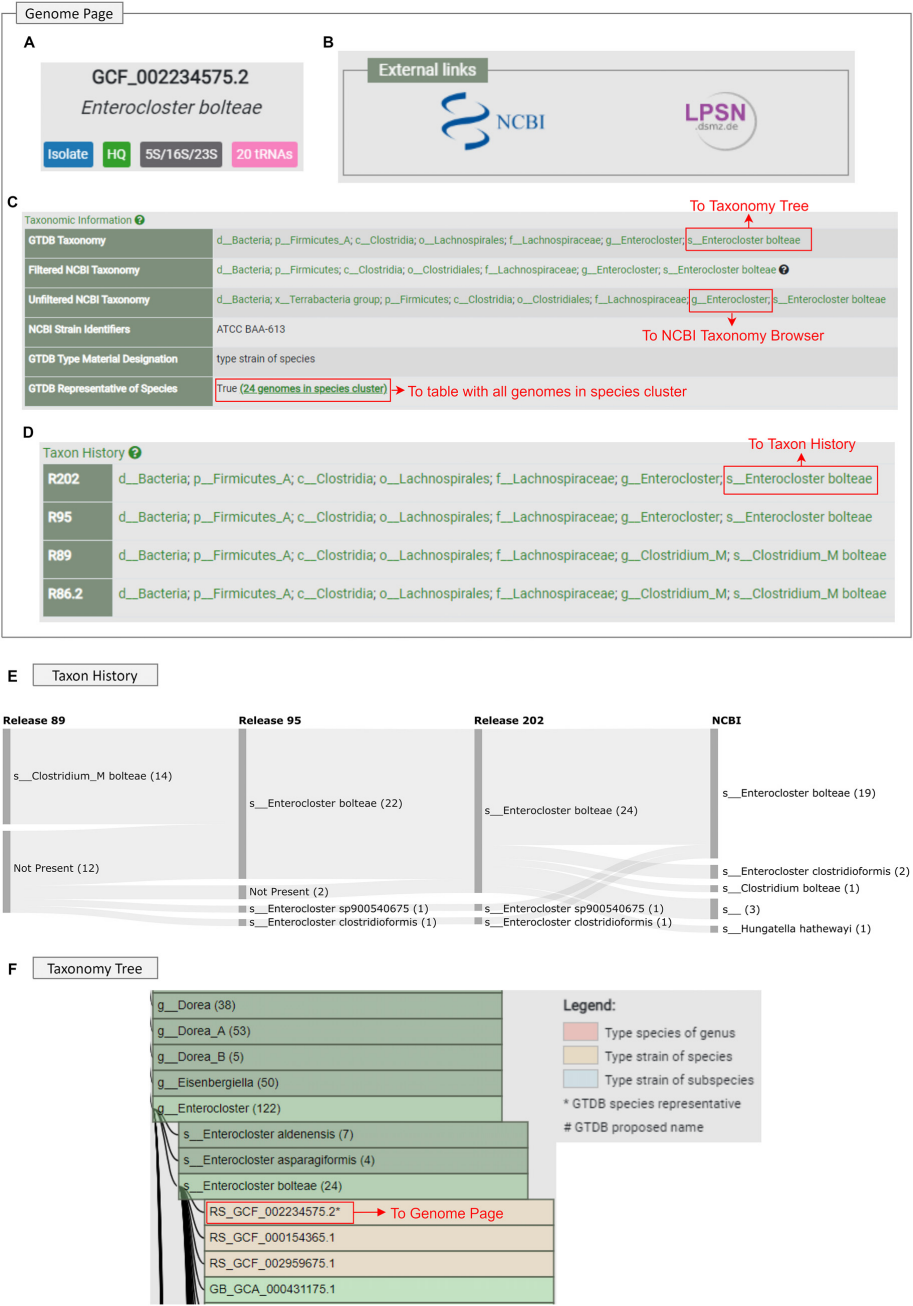
Taxonomic, nomenclatural, and assembly quality information provided for individual genomes. (**A**) NCBI genome assembly accession and GTDB quality badges associated with this genome. Hovering over a tag provides information about the criteria used to establish the tag. (**B**) An external link is always provided to the NCBI Assembly page of the genome as all GTDB genomes are sourced from NCBI. A link is also provided to an LPSN species page when a genome is established to be assembled from the type strain of a species based on LPSN nomenclatural information. (**C**) GTDB and NCBI classifications for this genome along with its strain identifiers, nomenclatural status at GTDB (i.e. type strain of the species), and GTDB species representative status. GTDB taxa link to their corresponding position in the GTDB Taxonomy Tree (i.e. Figure 2F) while NCBI taxa link to NCBI Taxonomy Browser pages. Each genome also links to a table indicating all genomes in the same GTDB species cluster. (**D**) GTDB classification of the genome in each GTDB release. GTDB taxa link to their corresponding Taxon History page (i.e. Figure 2E). (**E**) GTDB Taxon History view for genomes classified as *Enterocloster bolteae* indicating this species was reclassified from *Clostridium_M* to *Enterocloster* in GTDB R95. Numbers in parenthesis indicate the number of genomes assigned to a taxon. The Not Present label indicates genomes that were not available at the time of a GTDB release or failed the GTDB quality-control criteria used for the release, and thus had no GTDB classification. (**F**) GTDB Taxonomy Tree which provides a hierarchical exploration of the GTDB taxonomy and indicates nomenclatural type information, genomes selected as GTDB representatives, and Latin names in the GTDB which remain to be validated. Genomes link to their corresponding GTDB Genome page (i.e. A–D).

There have been a number of additional features and improvements to the GTDB website since its inception in November 2017, which we illustrate here using the *Enterocloster bolteae* genome GCF_002234575.2 (Figure 2). Each genome has an associated GTDB Genome page (Figure 2A–D) indicating taxonomic, nomenclatural, and assembly quality information. A badge system is used to provide a quick assessment of overall genome quality and indicate that GCF_002234575.2 is a high-quality (HQ) isolate genome assembly which contains the 5S, 16S, and 23S rRNA genes along with genes for all 20 tRNAs (Figure 2A). Additional assembly statistics such as CheckM completeness and contamination estimates (16), number of contigs, N50, genome size, protein count, and GC content are also provided on the Genome page (data not shown). A link to the NCBI Assembly page for GCF_002234575.2 is provided along with a link to the LPSN page for *E. bolteae* as this genome is recognized as being assembled from the type strain of the species according to LPSN (Figure 2B). Both GTDB and NCBI classifications are provided to allow for easy comparison between these taxonomic resources which have identical classifications for GCF_002234575.2 (Figure 2C). GCF_002234575.2 is annotated as being assembled from the strain ATCC BAA-613 at NCBI and considered to be assembled from the type strain of the species within the GTDB framework based on LPSN nomenclatural information for this species (Figure 2C). This genome has also been selected as the GTDB representative of the *E. bolteae* species cluster which comprises 24 genomes in GTDB R06-RS202 (Figure 2C).

We provide two avenues for exploring how GTDB classifications have changed between releases. At the bottom of each Genome page, the classification of the genome is provided for each GTDB release (Figure 2D). For example, GCF_002234575.2 was reclassified from *Clostridium_M bolteae* to *E. bolteae* in GTDB R95 in accordance with a new taxonomic opinion (17). Changes in GTDB classifications and their relationship to the NCBI taxonomy can also be explored at the taxon level using the GTDB Taxon History tool (Figure 2E). This visualization illustrates that *E. bolteae* was reclassified from *Clostridium_M* to *Enterocloster* in GTDB R05-RS95, that the number of genomes assigned to this species has steadily increased between releases, and that there are a number of incongruent classifications at NCBI including one genome assigned as *Clostridium bolteae* and another as *Hungatella hathewayi*.

The GTDB taxonomy can be explored hierarchically using the Taxonomy Tree tool (Figure 2F) or as an alphabetically sortable table (not shown). The tree view provides an approximation of the reference phylogenetic tree where taxa within a group are listed alphabetically with Latin names listed ahead of alphanumeric placeholder names. Type species of genera and type strains of species and subspecies are highlighted by color-coding. For example, there are three genomes assembled from the type strain of *E. bolteae* with the genome selected to represent this species in GTDB marked with an asterisk (Figure 2F) (5). GTDB includes over 400 Latin names that have been assigned by the GTDB curators but are still to be formally proposed (see *Policy Changes*). These are identified in the Taxonomy Tree with hashes and can also be marked and downloaded from the table view.

Searching functionality on the GTDB website has been expanded to allow for searches that are restricted to the GTDB taxonomy, NCBI taxonomy, NCBI organism name, or NCBI genome ID. This is useful for identifying genomes assigned to a specific GTDB or NCBI taxon. For example, this can be used to identify the 35 *Enterocloster clostridioformis* genomes in GTDB R06-RS202 without retrieving the two additional genomes with this classification at NCBI which are considered misclassified *E. bolteae* genomes in the GTDB (Figure 2E). An advanced search feature has been added to the GTDB website to allow specific subsets of genomes to be identified across all GTDB metadata fields. For example, this can be used to identify the 94 genomes in GTDB R06-RS202 which are classified as *Enterocloster* and have an estimated completeness >90%, estimated contamination <10%, and contain at least one 16S rRNA gene.

A primary goal of taxonomy is to aid in scientific communication. With this in mind, all GTDB web tools produce URLs which allow specific results to be communicated between researchers. The following URLs produce the Genome page, Taxonomy Tree view, Taxon History view, and described Advanced search results for *E. bolteae* (Figure 2):

- *Genome page:* https://gtdb.ecogenomic.org/genomes?gid=GCF_002234575.2
- *Taxonomy Tree*: https://gtdb.ecogenomic.org/tree?r=s__Enterocloster%20bolteae
- *Taxon History*: https://gtdb.ecogenomic.org/taxon_history/?from=R89&to=NCBI&query=s__Enterocloster%20bolteae
- *Advanced Search*: https://gtdb.ecogenomic.org/advanced?1=MX4yfmdfX0VudGVyb2Nsb3N0ZXI∼&3=Nn4xMn45MA∼∼&4=N34xMH4xMA∼∼&5=MTB.MTJ.MQ∼∼&exp=KDEmMyY0JjUp

## RESOURCE METHODOLOGY AND POLICIES

### Methodological updates

A number of methodological changes have occurred since the original description of the GTDB (4) including the addition of Archaea starting with GTDB R03-RS86 (18) and adoption of ANI-based genomic similarity criteria for delineating species in GTDB R04-RS89 (5), which is discussed in more detail below. The 120 bacterial and 122 archaeal marker genes used to infer the domain-specific GTDB reference trees have not changed but identification and alignment of these genes is performed using Pfam v33.1 instead of v27 (19) starting with R06-RS202. The archaeal tree was originally inferred with FastTree v2.1.10 (20) under the WAG + GAMMA model but has been inferred using IQ-TREE v1.6.9 (21) under the C10 + PMSF model as of GTDB R04-RS89 (18). The initial GTDB releases included in-house MAGs that at the time had not been deposited in an INSDC repository (1,4). However, starting with GTDB R05-RS95, the GTDB only incorporates genomes from the NCBI Assembly database (8) and explicitly excludes assemblies annotated as being from ‘large multi-isolate projects’ at NCBI as these comprise genomically well-represented species and would require substantial additional computational resources if considered. The methodology and reference data used for each release have been provided in the METHODS file starting with GTDB R04-RS89 (https://data.gtdb.ecogenomic.org/).

### Updating GTDB species clusters

Each GTDB species cluster is defined by a single representative genome and species assignments are established by considering the ANI and AF (alignment fraction) of genomes to these representatives as previously described (5). Here, we explain the methodology used to update these species clusters with each GTDB release. Species representatives are re-evaluated each release with an emphasis placed on retaining the same representative genomes for previously named species to preserve consistency between releases. However, the goal of stable representatives must be balanced with the utility of high-quality genomes as representatives, the need to incorporate changes in taxonomic opinion, and the need to correct genome classification or assembly errors.

**Figure 3.**
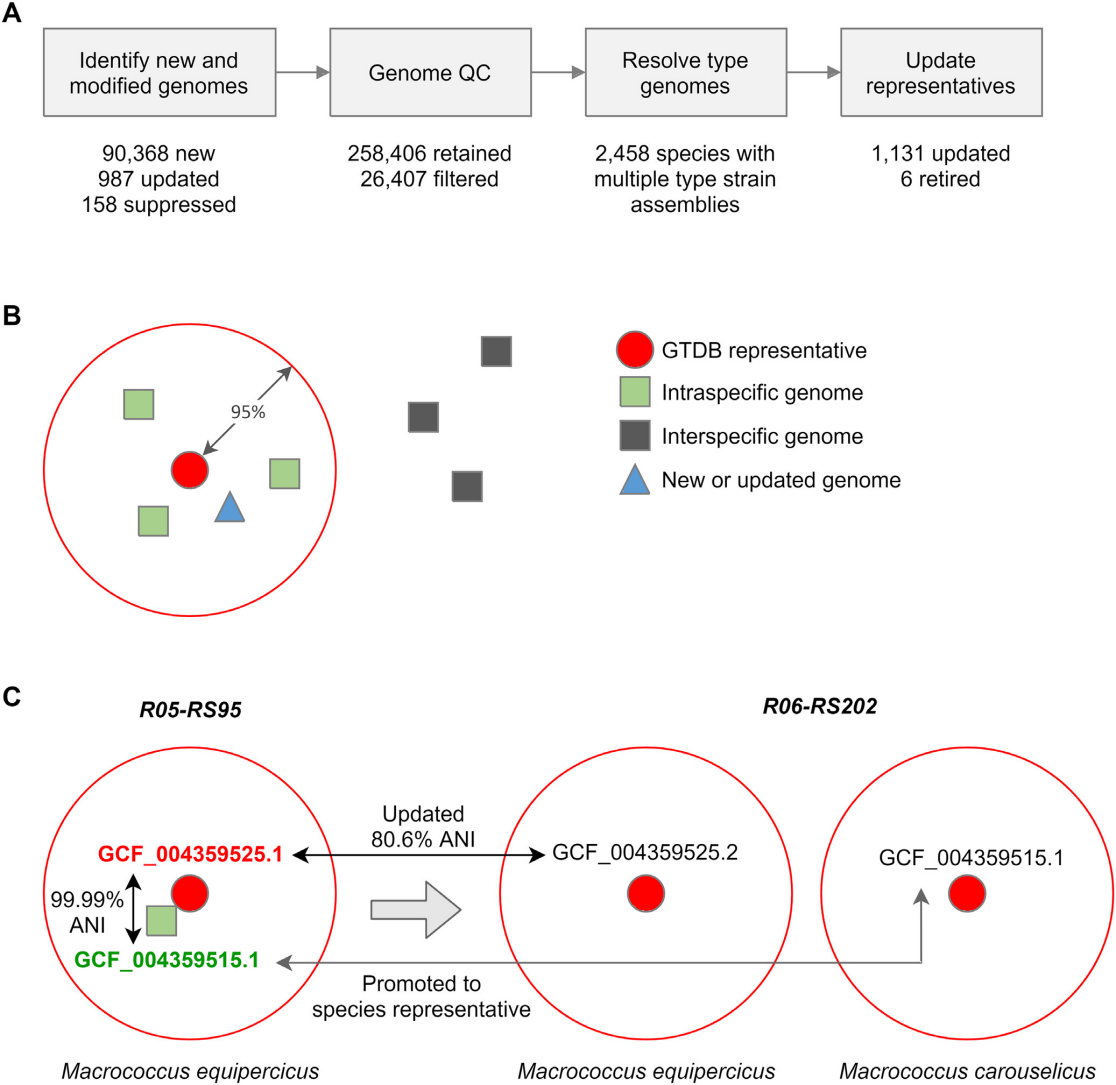
Updating species clusters with each GTDB release. (**A**) Workflow for updating GTDB species clusters with results for the most recent GTDB release, R06-RS202, given below each step. There were 90 368 new genomes in this release, 987 genomes where the assembly at NCBI was updated, and 158 genomes where the assembly was suppressed at NCBI and thus not used in this release. All genomes were subjected to quality control which resulted in 26 407 (9.3%) genomes being removed from consideration. There were 2,458 species where multiple genomes were identified as being assembled from the type strain of the species. Of these, 130 species had genomes that were sufficiently divergent to warrant manual inspection to establish the genome most likely to be from the type strain. The 31 910 representatives from the previous GTDB release, R05-RS95, were examined and 1131 (3.5%) updated to a new genome. In addition, 6 species defined in R05-RS95 were retired as the sole genome representing the species was suppressed at NCBI. (**B**) Illustrative example of a GTDB species cluster with previous and new genomes. Genomes are depicted by shapes and the distance between genomes scales with their ANI divergence. The large red circle indicates the ANI circumscription radii for assigning genomes to the current species clusters. The new/updated genome (blue triangle) will only replace the existing GTDB species representative (red circle) if the ANI between these genomes is sufficiently high and the new/updated genome is of sufficient quality (see Table 1). This decision is determined quantitatively using the balanced ANI score (see main text). (**C**) Updating the *Macrococcus equipercicus* species cluster from GTDB R05-RS95 to R06-RS202. The *M. equipercicus* genome assembly, GCF_004359525.1, was updated and found to be distinct from the previous assembly (ANI = 80.6%). Consequently, this genome formed a new species cluster and the genome GCF_004359515.1 was promoted to a species representative. GCF_004359525.2 is assembled from the type strain of *M. equipercicus* and GCF_004359515.1 assembled from the type strain of *M. carouselicus* indicating the *M. equipercicus* cluster in GTDB R05-RS95 actually represented the species *M. carouselicus* and was incorrectly classified as a result of the GCF_004359525.1 assembly being incorrect.

Updating GTDB species clusters consists of four steps (Figure 3A). Genomes in the current and previous releases are compared to identify new and updated genome assemblies, along with genomes that have been suppressed at NCBI indicating that they are no longer considered reliable (e.g. GCA_005039905.1). This is done by directly comparing the NCBI accession numbers (e.g. GCF_000267585.2) of the genomes in each release. Genomes of insufficient quality, as previously defined (5), are then identified and removed from further consideration. Next, species which contain multiple genomes identified as being assembled from the type strain of the species are examined. If any pair of these genomes has an ANI <99%, the genomes are manually inspected in order to establish which genome or genomes are most likely to represent the type strain of the species. Establishing the provenance of type material remains an ongoing challenge and this decision is made by considering a number of factors including the classification of 16S rRNA genes against the Living Tree Project database (13), the type status of genomes at NCBI, previous GTDB classifications, consideration of all pairwise ANI values and literature review.

GTDB representatives are updated according to two primary principles: (i) representatives should be assembled from the type strain of a species whenever possible, and (ii) representatives should only be replaced by equivalent assemblies of demonstrably higher quality (Figure 3B). These two principles are quantitatively defined by the Balanced ANI Score (BAS) which is 0.5 × (ANI score) + 0.5 × (quality score), where the ANI score is 100 – 20 × (100 – ANI to current representative) and the quality score is defined by the criteria given in Table 1. An existing representative is only replaced by a new or updated genome if it has a BAS ≥ 10 above the BAS of the current representative. This achieves the goal of taxonomic stability by requiring a new representative to be of increasingly higher quality (as defined by the quality score) the more dissimilar it is from the current representative (as defined by the ANI score).

**Table 1.** Criteria used to establish the quality score of genome assemblies

Criteria	Score
Assembled from type strain of species	1 000 000
Effective type strain of species according to NCBI	100 000
NCBI representative of species	10 000
Assembled from type strain of subspecies	1000
Complete genome	100
CheckM quality estimate	Completeness – 5 × contamination
MAG or SAG	–100
Contig count	–5 × (no. contigs/100)
Undetermined bases	–5 × (no. undetermined bases/10 000)
Full length 16S rRNA gene	10

There are two special cases that must be handled when updating species representatives. The first is when the assembly of an existing representative is updated. Although atypical, it is possible the new assembly will be sufficiently divergent from the previous assembly (e.g. by removal of a substantial amount of contamination) that a new representative should be selected. This is determined by calculating the BAS between all genomes in the species cluster, including the new assembly, to the previous assembly of the representative genome. The selected representative is the genome with the highest BAS which is typically the new assembly. The second special case occurs when a GTDB representative becomes suppressed at NCBI indicating that the assembly is no longer reliable. In such cases, the representative is replaced with the genome in the species cluster with the highest BAS to the suppressed representative in order to provide stability to the set of GTDB representatives to the extent possible. If the species cluster does not contain any genomes other than the suppressed representative, the species cluster is retired and will no longer appear in the GTDB.

GTDB species representatives have been largely stable since the introduction of ANI-based species clusters in RS04-RS89 with 96.7% of representatives being unchanged on average between releases (Table 2). This stability is particularly encouraging given the rapid growth in the number of GTDB species clusters (Figure 1C and D; Table 2). Numerous factors account for the changes that have occurred illustrating the benefits and need to regularly update taxonomic frameworks. Between R05-RS95 and R06-RS202 there were 111 representatives with an updated genome assembly at NCBI of which two changed sufficiently that a new GTDB representative was selected for the species cluster. For example, this occurred for GCF_004359525.1 as it has an ANI of only 80.6% to its updated assembly GCF_004359525.2, but the original species cluster was still deemed valid as the representative could be replaced with GCF_004359515.1 which is 99.99% similar to GCF_004359525.1 (Figure 3C). This discrepancy can be explained by the fact that GCF_004359525.2 is assembled from the type strain of *Macrococcus equipercicus* while GCF_004359515.1 is assembled from the type strain of *M. carouselicus* indicating that prior to the GCF_004359525.1 assembly being updated, an *M. carouselicus* strain was erroneously considered as the type strain for *M. equipercicus* at NCBI and in the GTDB. While the exception, such large-scale reassignments and assembly errors do occur and we make a best effort to identify and handle all such cases. There were 158 genomes in R06-RS202 which were removed from the GTDB as they were suppressed at NCBI. Among these 158 genomes, were 20 GTDB representatives which resulted in 14 species having new representatives selected and six species being retired as they were singleton clusters. A relatively larger number of changes were the result of replacing a non-type representative with a genome assembled from the type strain of the species (312 cases) or subspecies (7 cases). However, the majority of changes (595 cases) were the result of replacing a non-type representative with a higher-quality genome assembly with sufficiently high ANI to the existing representative (i.e. they satisfied the BAS replacement criterion).

**Table 2.** Updated GTDB species representatives and cluster assignments between releases

	*R04-RS89 to R05-RS95*	*R05-RS95 to R06-RS202*
***Species representative***	24 706 to 31 910	31 910 to 47 894
Unchanged	23 957 (96.97%)	30 773 (96.44%)
Changed	743 (3.01%)	1131 (3.54%)
***New species***	7218 (29.2% increase)	16 004 (50.2% increase)
***Genome cluster assignment***	145 896 to 194 600	194 600 to 258 406
Same cluster	145 566 (99.77%)	193 668 (99.52%)
Different cluster	172 (0.12%)	774 (0.40%)
Suppressed	158 (0.11%)	158 (0.08%)

After updating previous GTDB representatives, representatives for species with new effectively or validly published names must be selected and all remaining genomes are assigned to these species clusters or *de novo* species clusters using ANI to delineate species as previously described (5). Only 0.26% of genomes on average are assigned to a different species cluster between releases (Table 2). This is despite the assignment of genomes to species clusters needing to be determined *de novo* each release in order to account for new and modified GTDB species representatives. Such reassignments are the direct result of allowing representatives to change between releases but as illustrated above there are situations where new representatives must be selected (e.g. replacing GCF_004359525.1). We consider this small amount of reassignment acceptable as it allows genomes assembled from type strains and high-quality genome assemblies to become species representatives.

### Policy changes

The GTDB is an evolving resource that aims to balance continuity between releases with changes in classification methodology and taxonomic opinion to best serve the user community, which has necessitated a number of policy changes over time. As of R06-RS202, Latin names are no longer proposed by GTDB curators unless there is an associated publication with taxon descriptions (22,23). This change reflects our efforts to follow the International Code of Nomenclature of Prokaryotes (24) for determining correct names, including the proposal of *Candidatus* names. We currently discover new effectively published names by consulting the NCBI taxonomy and LPSN, receiving feedback from the community, and our own best efforts to read the literature. Effectively published Latin names above the rank of genus without designated type material, either a sequenced type strain or MAG, will no longer be incorporated into GTDB, and those that do will only be introduced when the associated type genome is present in GTDB. This change is necessary as establishing the correct interior node in the reference tree for taxa without type material can be ambiguous, particularly when the addition of new genomes or alternative inference methods results in the named taxon becoming polyphyletic in later releases. Latin names in preprints are also no longer incorporated into the GTDB even if their type genomes are already in the database as names can change between preprint and peer-reviewed publication (25,26). Finally, to preserve name continuity, we do not replace alphanumeric placeholder names with other placeholder names, even when names (mostly introduced in early releases) are unwieldy, e.g. CG2-30-70-394 which is used as a genus, family, order, class and phylum name. Placeholder names for new taxa in each release are now manually selected from a longer list of unique identifiers to avoid unwieldy names: NCBI organism name, NCBI infraspecific identifier, NCBI WGS identifier, or NCBI assembly identifier. We had previously aimed to release an update of the GTDB every six months and have averaged a new release every seven months. However, starting with R06-RS202 we plan to release updates every nine months in order to accommodate development of the GTDB between releases.

## AVERAGE NUCLEOTIDE IDENTITY FOR DELINEATING SPECIES

### Observations on the discreteness of ANI-based species in GTDB

An ANI threshold of 95–96% is widely considered the ‘gold standard’ for quantitatively delineating species (27–30) and it has recently been proposed that a genetic discontinuity exists between 83% and 95% ANI which would further support the use of ANI for defining species boundaries (31,32). This genetic discontinuity has also been observed between genomes recovered from specific environments such as the human gut and soil (33). However, when genome data are compared globally, i.e. not taking strain cohabitation into account, it is not uncommon for pairs of strains to have ANI values between 83% and 95% (5,34).

**Figure 4.**
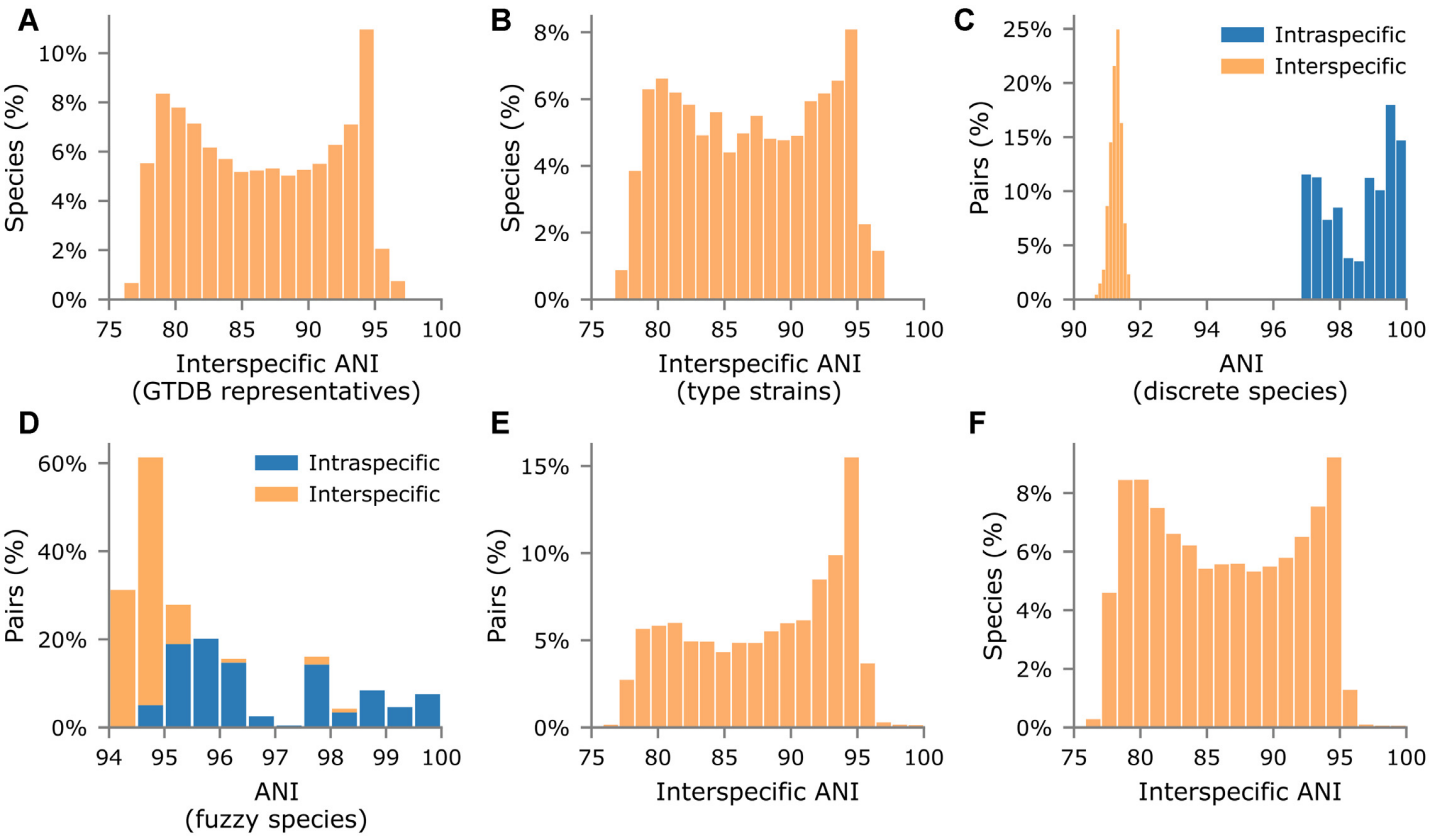
Use of genomic similarity for delineating species. (**A**) ANI values between 36 781 GTDB species representatives and their closest representative within the same genus. (**B**) Same plot as in A but restricted to the 9,687 species where the GTDB representative genome is assembled from the type strain. (**C**) Pairwise ANI between the 24 *Enterocloster bolteae* and 34 *E. clostridioformis* genomes in GTDB RS06-RS202. (**D**) Pairwise ANI between the 14 *Bradyrhizobium elkanii* and 8 *B. pachyrhizi* genomes in GTDB RS06-RS202. (**E**) ANI between genomes and their closest genome in a different, intrageneric species cluster (108 503 total pairs). (**F**) ANI between the closest pairs in plot E for each of the 35 147 species considered.

This issue is of interest as the GTDB uses a species definition based on the ANI to genomes selected as species representatives (5). Here we re-evaluated the evidence for a genetic discontinuity as the number of species and genomes within the GTDB has increased substantially since our original study. We demonstrate the lack of such a discontinuity by considering the ANI between closest pairs of representative genomes from GTDB species within the same genus (Figure 4A). If a meaningful discontinuity existed, we would expect to find few such pairs with an ANI between 83% and 95% supporting the idea of discrete, well-separated species boundaries. In contrast, we find nearly equal numbers of pairs with ANI values between 78% and 95%. The addition of non-representative genomes can only increase the likelihood of there being genomes with ANI values between 83% and 95% to a species representative and thus a genetic continuum between species. This lack of a genetic discontinuity also persists when considering only genomes assembled from the type strain of a species, indicating that this result is independent of the methodology used to select GTDB species representatives (Figure 4B). The above analyses do not speak to the possibility of discrete groups of strains defined by a large genetic discontinuity existing within specific environments as suggested by Olm *et al.* (33). However, further work is required to establish this discontinuity and specify how an environment-specific species definition might be used in practice.

While the above analysis indicates that there is no global genetic discontinuity between 83% and 95% ANI in the GTDB, it remains unclear as to whether strains generally form discrete, ANI-based species clusters. For example, the pairwise ANI values between *Enterocloster bolteae* and *E. clostridioformis* genomes form discrete clusters and have a bimodal distribution with a discontinuity between 92% and 96% ANI (Figure 4C). In contrast, pairwise ANI values between *Bradyrhizobium elkanii* and *B. pachyrhizi* genomes display a genetic continuum (Figure 4D) and can be described as fuzzy species (35). The discreteness of GTDB species clusters was assessed by considering the ANI between each genome and its closest genome in a different, intrageneric species cluster (see Methods). Discrete species will contain few or ideally no genomes with a high ANI to genomes from a different species. Disregarding GTDB species clusters delineated at an ANI >95% indicated that only 6.0% of the closest interspecific genome pairs have an ANI ≥ 95% and 80.8% of pairs are well-separated as indicated by having an ANI ≤94% (Figure 4E). In terms of species, 2.2% can be described as fuzzy as they have one or more genomes with an interspecific ANI value ≥95% and 89.5% can be described as discrete as they contain no genomes with an interspecific ANI value >94% (Figure 4F). Encouragingly, this indicates that GTDB species clusters are largely discrete, but this result should be interpreted with care as most bacterial and archaeal species lack genomic representation based on even conservative estimates of the number of prokaryotic species in nature (36).

The use of ANI to delineate species despite the lack of clear evidence for discrete species boundaries in the GTDB dataset is a pragmatic approach for organizing the rapidly growing biodiversity being discovered with metagenomic approaches (2,3,15). Leaving this diversity unclassified would severely limit the ability to effectively communicate scientific results as exemplified by 28.5% of genomes at NCBI lacking a species classification and 77.0% of GTDB R06-RS202 species clusters having an alphanumeric placeholder name. Doolittle and Papke (37) best articulate the rationale for a pragmatic species definition: ‘What we want from a species definition is a set of easily applied and stable rules by which to decide when two organisms are similar enough in their genomic and/or phenotypic properties to be given the same name. The needs for such a guide to taxonomic practice in medicine, biotechnology and defense are obvious, and even arbitrary rules to satisfy them would be better than no rules at all.’

## CONCLUDING REMARKS AND FUTURE PLANS

GTDB is in active development and there are a number of initiatives underway to make this resource more comprehensive and accurate. As MAGs are not always submitted to INSDC repositories, we are exploring incorporation of additional repositories into the GTDB such as MGnify (38). The quality of MAGs is an ongoing concern and we are evaluating methods for decontaminating MAGs (39) and identifying and removing MAGs that may contribute to phylogenetic instability (40). Related to MAG quality, we expect to change the AF criteria used for assigning genomes to GTDB species clusters from 0.65 to 0.5 starting in the next GTDB release in order to better accommodate the growing number of incomplete MAGs contained in the GTDB which can cause the AF between MAGs to be artificially low. There is also an ongoing need to evaluate best practices in terms of tree inference methods, which has been initially explored for the archaeal tree and resulted in the adoption of IQ-TREE for this domain. We envisage that further evaluations will result in modifications to the bacterial and archaeal marker sets in future GTDB releases. Feedback is essential for the GTDB to best serve the community and we encourage suggestions and comments on either the GTDB Forum (https://forum.gtdb.ecogenomic.org/) or directly to the GTDB development team (https://gtdb.ecogenomic.org/about).

## METHODS

ANI and AF values were calculated with FastANI v1.3 (31). The discreteness of GTDB species clusters was assessed by identifying the closest genome to a genome within a different, intrageneric species cluster. The 35 147 species in genera with multiple species and with an ANI circumscription radius of 95% were considered in this analysis (species with an ANI radius >95% are exceptional cases in the GTDB and result from another species being within 95% ANI that was retained in the GTDB to preserve historical classifications). In order to reduce computational requirements, it was assumed that if the closest GTDB representative genome for species A was found to be species B, that the closest genome for any genome in species A will be in species B. Furthermore, for the 87 species containing >150 genomes only a randomly selected subset of 150 genomes was considered though these were compared to all genomes in species B regardless of the number of genomes in this species.

## DATA AVAILABILITY

The GTDB can be accessed at https://gtdb.ecogenomic.org/ and data files for each GTDB release are available from https://data.gtdb.ecogenomic.org/. Genomes comprising the GTDB are obtained from the NCBI Assembly database (www.ncbi.nlm.nih.gov/assembly). GTDB species clusters are updated using the Python code at https://github.com/Ecogenomics/gtdb-species-clusters which is made available under the GNU GPL v3.0.

## Supplementary Material

gkab776_Supplemental_File

## References

[1] Parks D.H. , Rinke C., Chuvochina M., Chaumeil P.-A., Woodcroft B.J., Evans P.N., Hugenholtz P., Tyson G.W. Recovery of nearly 8,000 metagenome-assembled genomes substantially expands the tree of life. Nat. Microbiol. 2017; 2:1533–1542. 28894102 10.1038/s41564-017-0012-7

[2] Pasolli E. , Asnicar F., Manara S., Zolfo M., Karcher N., Armanini F., Beghini F., Manghi P., Tett A., Ghensi P. et al. Extensive unexplored human microbiome diversity revealed by over 150,000 genomes from metagenomes spanning age, geography, and lifestyle. Cell. 2019; 176:649–662. 30661755 10.1016/j.cell.2019.01.001 PMC6349461

[3] Nayfach S. , Roux S., Seshadri R., Udwary D., Varghese N., Schulz F., Wu D., Paez-Espino D., Chen I.-M., Huntemann M. et al. A genomic catalog of Earth's microbiomes. Nat. Biotechnol. 2020; 39:499–509. 33169036 10.1038/s41587-020-0718-6 PMC8041624

[4] Parks D.H. , Chuvochina M., Waite D.W., Rinke C., Skarshewski A., Chaumeil P.-A., Hugenholtz P. A standardized bacterial taxonomy based on genome phylogeny substantially revises the tree of life. Nat. Biotechnol. 2018; 36:996–1004. 30148503 10.1038/nbt.4229

[5] Parks D.H. , Chuvochina M., Chaumeil P.-A., Rinke C., Mussig A.J., Hugenholtz P. A complete domain-to-species taxonomy for Bacteria and Archaea. Nat. Biotechnol. 2020; 38:1079–1086. 32341564 10.1038/s41587-020-0501-8

[6] Chaumeil P.A. , Mussig A.J., Hugenholtz P., Parks D.H. GTDB-Tk: A toolkit to classify genomes with the genome taxonomy database. Bioinformatics. 2020; 36:1925–1927. 10.1093/bioinformatics/btz848 PMC7703759 31730192

[7] Schoch C.L. , Ciufo S., Domrachev M., Hotton C.L., Kannan S., Khovanskaya R., Leipe D., Mcveigh R., O’Neill K., Robbertse B. et al. NCBI Taxonomy: a comprehensive update on curation, resources and tools. Database. 2020; 2020:baaa062. 32761142 10.1093/database/baaa062 PMC7408187

[8] Kitts P.A. , Church D.M., Thibaud-Nissen F., Choi J., Hem V., Sapojnikov V., Smith R.G., Tatusova T., Xiang C., Zherikov A. et al. Assembly: a resource for assembled genomes at NCBI. Nucleic Acids Res. 2016; 44:D73–D80. 26578580 10.1093/nar/gkv1226 PMC4702866

[9] Arita Masanori , Karsch-Mizrachi Ilene, Cochrane Guy The international nucleotide sequence database collaboration. Nucleic Acids Res. 2021; 49:D121–D124. 33166387 10.1093/nar/gkaa967 PMC7778961

[10] Fukuda A. , Kodama Y., Mashima J., Fujisawa T., Ogasawara O. DDBJ update: streamlining submission and access of human data. Nucleic Acids Res. 2021; 49:D71. 33156332 10.1093/nar/gkaa982 PMC7779041

[11] Cantelli G. , Cochrane G., Brooksbank C., McDonagh E., Flicek P., McEntyre J., Birney E., Apweiler R. The European Bioinformatics Institute: empowering cooperation in response to a global health crisis. Nucleic Acids Res. 2021; 49:D29. 33245775 10.1093/nar/gkaa1077 PMC7778996

[12] Parte A.C. , Carbasse J.S., Meier-Kolthoff J.P., Reimer L.C., Göker M. List of prokaryotic names with standing in nomenclature (LPSN) moves to the DSMZ. Int. J. Syst. Evol. Microbiol. 2020; 70:5607–5612. 32701423 10.1099/ijsem.0.004332 PMC7723251

[13] Yilmaz P. , Parfrey L.W., Yarza P., Gerken J., Pruesse E., Quast C., Schweer T., Peplies J., Ludwig W., Glöckner F.O. The SILVA and “All-species Living Tree Project (LTP)” taxonomic frameworks. Nucleic Acids Res. 2014; 42:D643. 24293649 10.1093/nar/gkt1209 PMC3965112

[14] Li W. , O’Neill K.R., Haft D.H., DiCuccio M., Chetvernin V., Badretdin A., Coulouris G., Chitsaz F., Derbyshire M.K., Durkin A.S. et al. RefSeq: expanding the Prokaryotic Genome Annotation Pipeline reach with protein family model curation. Nucleic Acids Res. 2021; 49:D1020. 33270901 10.1093/nar/gkaa1105 PMC7779008

[15] Almeida A. , Mitchell A.L., Boland M., Forster S.C., Gloor G.B., Tarkowska A., Lawley T.D., Finn R.D. A new genomic blueprint of the human gut microbiota. Nature. 2019; 568:499–504. 30745586 10.1038/s41586-019-0965-1 PMC6784870

[16] Parks D.H. , Imelfort M., Skennerton C.T., Hugenholtz P., Tyson G.W. CheckM: assessing the quality of microbial genomes recovered from isolates, single cells, and metagenomes. Genome Res. 2015; 25:1043–1055. 25977477 10.1101/gr.186072.114 PMC4484387

[17] Haas K.N. , Blanchard J.L. Reclassification of the Clostridium clostridioforme and Clostridium sphenoides clades as Enterocloster gen. Nov. and Lacrimispora gen. nov., including reclassification of 15 taxa. Int. J. Syst. Evol. Microbiol. 2020; 70:23–34. 31782700 10.1099/ijsem.0.003698

[18] Rinke C. , Chuvochina M., Mussig A.J., Chaumeil P.-A., Davín A.A., Waite D.W., Whitman W.B., Parks D.H., Hugenholtz P. A standardized archaeal taxonomy for the Genome Taxonomy Database. Nat. Microbiol. 2021; 6:946–959. 34155373 10.1038/s41564-021-00918-8

[19] Finn R.D. , Bateman A., Clements J., Coggill P., Eberhardt R.Y., Eddy S.R., Heger A., Hetherington K., Holm L., Mistry J. et al. Pfam: the protein families database. Nucleic Acids Res. 2014; 42:D222. 24288371 10.1093/nar/gkt1223 PMC3965110

[20] Price M.N. , Dehal P.S., Arkin A.P. FastTree: computing large minimum evolution trees with profiles instead of a distance matrix. Mol. Biol. Evol. 2009; 26:1641–1650. 19377059 10.1093/molbev/msp077 PMC2693737

[21] Nguyen L.T. , Schmidt H.A., Haeseler A., Minh B.Q. IQ-TREE: a fast and effective stochastic algorithm for estimating maximum-likelihood phylogenies. Mol. Biol. Evol. 2015; 32:268–274. 25371430 10.1093/molbev/msu300 PMC4271533

[22] Chuvochina M. , Rinke C., Parks D.H., Rappé M.S., Tyson G.W., Yilmaz P., Whitman W.B., Hugenholtz P. The importance of designating type material for uncultured taxa. Syst. Appl. Microbiol. 2019; 42:15–21. 30098831 10.1016/j.syapm.2018.07.003

[23] Waite D.W. , Chuvochina M., Pelikan C., Parks D.H., Yilmaz P., Wagner M., Loy A., Naganuma T., Nakai R., Whitman W.B. et al. Proposal to reclassify the proteobacterial classes Deltaproteobacteria and Oligoflexia, and the phylum Thermodesulfobacteria into four phyla reflecting major functional capabilities. Int. J. Syst. Evol. Microbiol. 2020; 70:5972–6016. 33151140 10.1099/ijsem.0.004213

[24] Parker C.T. , Tindall B.J., Garrity G.M. International code of nomenclature of prokaryotes: prokaryotic code (2008 revision). Int. J. Syst. Evol. Microbiol. 2019; 69:S1–S111. 26596770 10.1099/ijsem.0.000778

[25] Tschoeke D. , Vidal L., Campeão M., Salazar V.W., Swings J., Thompson F., Thompson C. Unlocking the genomic taxonomy of the Prochlorococcus collective. 2020; bioRxiv doi:12 March 2020, preprint: not peer reviewed 10.1101/2020.03.09.980698. 32468160

[26] Tschoeke D. , Salazar V.W., Vidal L., Campeão M., Swings J., Thompson F., Thompson C. Unlocking the genomic taxonomy of the Prochlorococcus collective. Microb. Ecol. 2020; 80:546–558. 32468160 10.1007/s00248-020-01526-5

[27] Konstantinidis K.T. , Tiedje J.M. Genomic insights that advance the species definition for prokaryotes. Proc. Natl. Acad. Sci. U.S.A. 2005; 102:2567. 15701695 10.1073/pnas.0409727102 PMC549018

[28] Richter M. , Rosselló-Móra R. Shifting the genomic gold standard for the prokaryotic species definition. Proc. Natl. Acad. Sci. U.S.A. 2009; 106:19126–19131. 19855009 10.1073/pnas.0906412106 PMC2776425

[29] Ciufo S. , Kannan S., Sharma S., Badretdin A., Clark K., Turner S., Brover S., Schoch C.L., Kimchi A., DiCuccio M. Using average nucleotide identity to improve taxonomic assignments in prokaryotic genomes at the NCBI. Int. J. Syst. Evol. Microbiol. 2018; 68:2386–2392. 29792589 10.1099/ijsem.0.002809 PMC6978984

[30] Chun J. , Oren A., Ventosa A., Christensen H., Arahal D.R., da Costa M.S., Rooney A.P., Yi H., Xu X.-W., de Meyer S. et al. Proposed minimal standards for the use of genome data for the taxonomy of prokaryotes. Int. J. Syst. Evol. Microbiol. 2018; 68:461–466. 29292687 10.1099/ijsem.0.002516

[31] Jain C. , Rodriguez-R L.M., Phillippy A.M., Konstantinidis K.T., Aluru S. High throughput ANI analysis of 90K prokaryotic genomes reveals clear species boundaries. Nat. Commun. 2018; 9:5114. 30504855 10.1038/s41467-018-07641-9 PMC6269478

[32] Rodriguez-R L.M. , Jain C., Conrad R.E., Aluru S., Konstantinidis K.T. Reply to: “Re-evaluating the evidence for a universal genetic boundary among microbial species”. Nat. Commun. 2021; 12:4060. 34234115 10.1038/s41467-021-24129-1 PMC8263725

[33] Olm M.R. , Crits-Christoph A., Diamond S., Lavy A., Matheus Carnevali P.B., Banfield J.F. Consistent metagenome-derived metrics verify and delineate bacterial species boundaries. mSystems. 2020; 5:e00731-19. 31937678 10.1128/mSystems.00731-19 PMC6967389

[34] Murray C.S. , Gao Y., Wu M. Re-evaluating the evidence for a universal genetic boundary among microbial species. Nat. Commun. 2021; 12:4059. 34234129 10.1038/s41467-021-24128-2 PMC8263626

[35] Hanage W.P. , Fraser C., Spratt B.G. Fuzzy species among recombinogenic bacteria. BMC Biol. 2005; 3:6. 15752428 10.1186/1741-7007-3-6 PMC554772

[36] Louca S. , Mazel F., Doebeli M., Parfrey L.W. A census-based estimate of Earth's bacterial and archaeal diversity. PLoS Biol. 2019; 17:e3000106. 30716065 10.1371/journal.pbio.3000106 PMC6361415

[37] Doolittle W.F. , Papke R.T. Genomics and the bacterial species problem. Genome Biol. 2006; 7:116. 17020593 10.1186/gb-2006-7-9-116 PMC1794555

[38] Mitchell A.L. , Almeida A., Beracochea M., Boland M., Burgin J., Cochrane G., Crusoe M.R., Kale V., Potter S.C., Richardson L.J. et al. MGnify: the microbiome analysis resource in 2020. Nucleic Acids Res. 2020; 48:D570–D578. 31696235 10.1093/nar/gkz1035 PMC7145632

[39] Orakov A. , Fullam A., Coelho L.P., Khedkar S., Szklarczyk D., Mende D.R., Schmidt T.S.B., Bork P. GUNC: detection of chimerism and contamination in prokaryotic genomes. Genome Biol. 2021; 22:178. 34120611 10.1186/s13059-021-02393-0 PMC8201837

[40] Aberer A.J. , Krompass D., Stamatakis A. Pruning rogue taxa improves phylogenetic accuracy: an efficient algorithm and webservice. Syst. Biol. 2013; 62:162–166. 22962004 10.1093/sysbio/sys078 PMC3526802

