# Supplementary material for: GTDB: an ongoing census of bacterial and archaeal diversity through a phylogenetically consistent, rank normalized and complete genome-based taxonomy
Source: Nucleic Acids Res. 2021 Sep 14;50(D1):D785–94. doi: 10.1093/nar/gkab776 (PMC8728215; doi:10.1093/nar/gkab776)
Supplement: gkab776_Supplemental_File [file gkab776_supplemental_file.pdf]

GTDB: an ongoing census of bacterial and archaeal diversity through a phylogenetically consistent, rank normalized, and complete genome-based taxonomy

Donovan H Parks, Maria Chuvochina, Christian Rinke, Aaron J Mussig, Pierre-Alain Chaumeil, Philip Hugenholtz

Supplementary Table 1. Growth in number of genomes and taxa in the GTDB.

	R01-RS80	*R02-RS83*	*R03-RS86.2*	*R04-RS89*	*R05-RS95*	*R06-RS202*
**Release data**	1-Nov-17	8-Mar-18	19-Aug-18	17-Jun-19	17-Jul-20	27-Apr-21
**Bacterial genomes**	94759	109419	125243	143512	191527	254090
Isolates	80730	95056	108974	122939	164719	202676
MAGs	13839	14166	16070	19880	26107	50669
SAGs	190	197	199	693	701	745
**Archaeal genomes**	n/a	n/a	2075	2392	3073	4316
Isolates	n/a	n/a	779	777	946	1195
MAGs	n/a	n/a	1269	1574	2079	3068
SAGs	n/a	n/a	27	41	48	53
**Bacterial taxa**						
Phyla	98	99	104	112	111	127
Classes	262	269	284	296	327	360
Orders	702	728	773	816	917	1163
Families	1558	1624	1809	1969	2282	2886
Genera	5426	5697	6482	7372	8778	12037
Species	8885	9912	11043	23458	30238	45555
**Archaeal taxa**						
Phyla	n/a	n/a	11	16	18	19
Classes	n/a	n/a	39	36	42	47
Orders	n/a	n/a	96	96	103	116
Families	n/a	n/a	188	238	276	336
Genera	n/a	n/a	440	534	650	851
Species	n/a	n/a	510	1248	1672	2339

Supplementary Table 2. Species with the largest number of genomes in the GTDB since the inception of ANI-based species clusters in R04-RS89.

	R04-RS89	*R05-RS95*	*R06-RS202*
Escherichia flexneri	9,084	12,267	14,743
Staphylococcus aureus	9,444	10,497	12,022
Salmonella enterica	8,698	10,510	11,523
Klebsiella pneumoniae	4,086	7,580	9,621
Streptococcus pneumoniae	8,201	8,340	8,405
Mycobacterium tuberculosis	5,596	6,514	6,684
Pseudomonas aeruginosa	2,744	4,561	5,211
Acinetobacter baumannii	2,796	3,975	4,766
Escherichia coli	2,432	3,724	4,370
Streptococcus pyogenes	427	1,915	2,136
Clostridioides difficile	1,079	2,007	2,079
Mycobacterium abscessus	1,589	1,673	1,686
Total	56,176	73,563	83,246
Growth	-	31.0%	13.1%
